# Versatile Vessel-on-a-Chip Platform for Studying Key Features of Blood Vascular Tumors

**DOI:** 10.3390/bioengineering8060081

**Published:** 2021-06-09

**Authors:** Marina Llenas, Roberto Paoli, Natalia Feiner-Gracia, Lorenzo Albertazzi, Josep Samitier, David Caballero

**Affiliations:** 1 Institute for Bioengineering of Catalonia (IBEC), The Barcelona Institute of Science and Technology (BIST), Baldiri Reixac 15–21, 08028 Barcelona, Spain; mllenas@icmab.es (M.L.); rpaoli@ibecbarcelona.eu (R.P.); nfeinerg@gmail.com (N.F.-G.); 2 Department of Electronics and Biomedical Engineering, University of Barcelona, C. Martí i Franqués 1, 08028 Barcelona, Spain; 3 Centro de Investigación Biomédica en Red en Bioingeniería, Biomateriales y Nanomedicina (CIBER-BBN), 28029 Madrid, Spain; 4 Department of Biomedical Engineering and Institute of Complex Molecular Systems (ICMS), Eindhoven University of Technology, 5612AZ Eindhoven, The Netherlands

**Keywords:** in vitro model, microfluidics, organ-on-chip, vascular tumor, vessel

## Abstract

Tumor vessel-on-a-chip systems have attracted the interest of the cancer research community due to their ability to accurately recapitulate the multiple dynamic events of the metastatic cascade. Vessel-on-a-chip microfluidic platforms have been less utilized for investigating the distinctive features and functional heterogeneities of tumor-derived vascular networks. In particular, vascular tumors are characterized by the massive formation of thrombi and severe bleeding, a rare and life-threatening situation for which there are yet no clear therapeutic guidelines. This is mainly due to the lack of technological platforms capable of reproducing these characteristic traits of the pathology in a simple and well-controlled manner. Herein, we report the fabrication of a versatile tumor vessel-on-a-chip platform to reproduce, investigate, and characterize the massive formation of thrombi and hemorrhage on-chip in a fast and easy manner. Despite its simplicity, this method offers multiple advantages to recapitulate the pathophysiological events of vascular tumors, and therefore, may find useful applications in the field of vascular-related diseases, while at the same time being an alternative to more complex approaches.

## 1. Introduction

### 1.1. The Unique Characteristics of the Human Tumor Vasculature

The human vasculature is a vital system for the continuous circulation of blood, nutrients, and gases throughout the body. Healthy vasculature has a well-defined hierarchical structure organized in networks of evenly spaced arteries, arterioles, capillaries, venules, and veins. In contrast, during pathological processes, such as tumor development, the vascular system forms abnormal vessel architectures [1]. Additionally, the enhanced fenestrated endothelium alters the normal flow patterns in the vessels facilitating the invasion of cancer cells. Vessel anomalies can also originate from the so-called vascular tumors, which are neoplastic transformations of the blood vessels [2]. In this type of tumor, the vessels are unevenly distributed exhibiting abnormal serpentine-like morphologies with loosely interconnected cells that cause functional heterogeneities in the blood flow (Figure S1) [1]. There is an increasing interest in the scientific community in the development of innovative technological platforms to recapitulate the distinctive features of vascular tumors. Typically, conventional (static) in vitro models of the vasculature have been utilized. However, these platforms are nowadays considered obsolete since they are unable to reproduce the complex dynamic microenvironment and, importantly, the pathological events occurring inside the vasculature. Similarly, in vivo models, such as Zebrafish or mice, are technically more challenging to manipulate, and are associated with serious ethical and economic concerns. Finally, the*—in ovo/ex ovo—*chick chorioallantoic membrane (CAM) is a well-established and low-cost assay used for the study of vascularization, but has a limited access and low survivability. To overcome these limitations, vessel-on-a-chip platforms have emerged as physiologically relevant systems capable of reproducing the functional units and dynamics of the native vasculature within a microfluidic chip [3,4]. One of the most advantageous features of this microfluidic-based technology is its capability to control physiological flow levels, shear stress, cell content, and chip architecture [5,6,7]. Indeed, vessel- (or vascular-/capillary-) on-a-chip models have been widely reported for studying blood vessel development, function, and pathogenesis [8,9]. Due to its simplicity, standard replica molding and soft lithography have been typically used to reproduce the native geometry of microvascular structures. The resulting micro-sized hollow vessel-like channels are usually coated with adhesive proteins of the extracellular matrix (ECM) and lined with endothelial cells (e.g., HUVEC) [10,11]. Despite using solid materials and failing to recapitulate key features occurring in the native tumor microenvironment, lining microfabricated channels with endothelial cells is still widely employed in cancer research as a versatile model to investigate a myriad of pathophysiological events.

### 1.2. Vessel-On-A-Chip for Reproducing Distinctive Features of Rare Vascular Tumors

Vascular tumors formed from blood (e.g., hemangioma) or lymph (e.g., lymphangiomas) vessels can be either benign or malignant [12]. The former is the most common benign tumors of infancy that appears early after birth (4–5% prevalence). Malignant—very aggressive—ones include epithelioid hemangioendothelioma or angiosarcoma of soft tissue [13]. The symptoms of vascular tumors are very heterogeneous, ranging from mild tissue defect or chronic pain to more serious and life-threatening coagulopathies, such as thrombi formation or bleeding (hemorrhage). Strikingly, there are still no well-established therapeutic guidelines to treat them, in part due to the lack of technological platforms capable of reproducing the distinctive features of the pathology in a simple and well-controlled manner. In this regard, vessel-on-a-chip models can provide a realistic and dynamic microenvironment to investigate the characteristic events of vascular tumors. Herein, we report the fabrication and characterization of a versatile tumor vessel-on-a-chip platform to investigate the hemodynamics and key traits of—infantile—vascular tumors. In particular, we describe the massive formation of clot-like aggregates and barrier-like disruption, thus reproducing physiological thrombi and hemorrhage on-chip, a distinctive feature of rare subtypes of vascular tumors. Despite its simplicity, this vessel-on-a-chip offers certain advantages compared to more complex systems for reproducing certain pathophysiological events of vascular tumors in a fast and simple manner, and therefore, may be useful to, e.g., assess the effect of pharmacological compounds in rapid and systematic manner. Finally, and importantly, it is not our aim to develop an accurate and realistic model of pediatric vascular tumors or to investigate in detail the etiology of the disease, but to develop a versatile platform that has the ability to easily reproduce particular traits of the disease.

## 2. Material and Methods

*Fabrication of the microfluidic vessel-on-a-chip:* Standard UV-photolithography and soft lithography were used to fabricate the microfluidic device. Briefly, a microfluidic channel with a serpentine morphology and rectangular cross-section (200 μm × 50 μm; width × height) was designed with Computer-aided design (CAD) software mimicking the tortuous architecture reminiscent of vascular tumors, and reproduced in an acetate photomask. Next, SU8-2050 (Microchem) photoresine (Round Rock, TX, USA) was spin coated on top of a Si wafer at 3000 rpm to obtain a thickness of 50 μm. A mask aligner (SÜSS Microtec, MJ84, Garching, Germany) was used to UV-irradiate the photoresine-coated wafer. Non-exposed regions were removed (SU-8 developer, Michochem, Round Rock, TX, USA) The resulting microchannel mold was then silanized with trichlorosilane (Sigma) by vapor phase for 1 h, and cured at 70 °C for 4 h. Next, the mold was replicated in polydimethylsiloxane (PDMS) by mixing the cross-linker and the pre-polymer (Sylgard 184, Dow Corning) in a 1:10 (*w*/*w*) ratio. After degassing and curing (2 h, 65 °C), the PDMS replica was carefully peeled off, perforated for creating the inlet/outlets, and bonded to a glass slide by O_2_ plasma for 30 s (Harrick).

**Figure 1:**
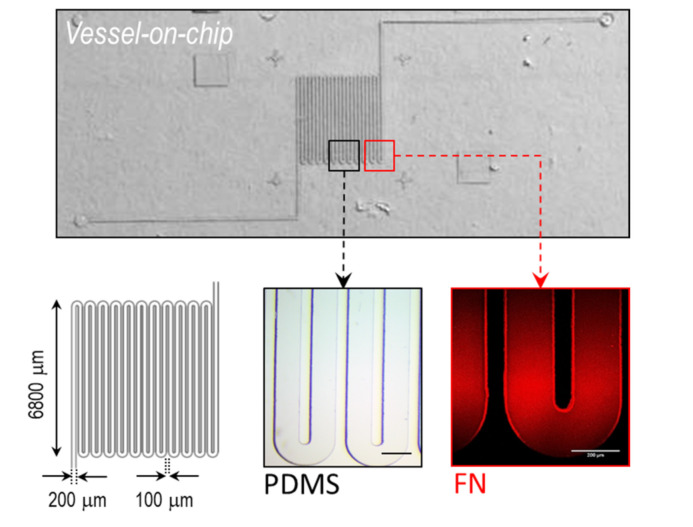
Microfluidic vessel-on-a-chip. (**Upper**) Optical microscopy image of the polydimethylsiloxane (PDMS) microfluidic chip. (**Lower**) Left—Scheme showing the dimensions of the microchannel; middle and right—magnified image of the fabricated and fibronectin-coated (rhodamine) channel. Scale bar: 200 μm.

*Microchannel functionalization:* Fibronectin (20 μg mL^−1^; Sigma) PBS 1× solution from bovine plasma was injected inside the O_2_ plasma-activated microchannel under low flow conditions for 15 min. The flow was stopped to promote protein adhesion for 5 min. During the functionalization process, the chip was stored at 37 °C to facilitate protein adhesion. This process was repeated thrice. Finally, the microchannel was washed with sterile PBS 1× and stored at 4 °C in the dark prior to use. To confirm protein adhesion, rhodamine-labeled fibronectin was used (Cytoskeleton, Inc. Denver, CO, USA) (Figure 1).

*Optical microscopy:* An inverted optical microscope (Olympus IX71, Olympus, Tokyo, Japan) with a 4× and 20× phase-contrast air objective was used for real-time imaging (acquisition rate: 1 image/2 ms). The microscope was equipped with a charge-coupled device camera (Hamamatsu, Shizuoka, Japan), an Olympus Hg lamp (Olympus, Tokyo, Japan), and a red filter (Thorlabs, Newton, NJ, USA) to prevent phototoxicity. An environmental chamber (Okolab, Pozzuoli, Italy) maintaining physiological conditions (37 °C, 5% CO_2_) was used for experiments with cells.

*Cell culture:* Human umbilical vein endothelial cells (HUVEC; Millipore) were cultured in EndoGRO medium (EndoGRO-LS complete media kit, SCME001, Millipore) in a T75 culture flask as recommended by the supplier. The culture media contained basal medium, EndoGRO-LS supplement (0.2%), rh-EGF (0.1%), ascorbic acid (0.1%), L-Glutamine (5%), hydrocortisone hemisuccinate (0.1%), heparine sulfate (0.1%) and FBS (2%). The culture media did not contain antimicrobials and phenol red. For cell seeding inside the freshly fibronectin-coated microfluidic channel, cells were rinsed with PBS 1× and trypsinized (Trypsin 0.25 EDTA; Life Technologies). After 5 min in the incubator, the trypsin was neutralized with medium, cells collected, and centrifuged at 2000 rpm for 5 min. For the experiments, 2.5 × 10^6^ cells were re-suspended in 100 μL and injected manually inside the microchannel applying a mild pressure. After 1 h, the chip was flipped and additional 2.5 × 10^6^ cells were injected to ensure the homogenous coating of the channel. During this process, the chip was kept in the incubator at 5% CO_2_ and 37 °C. After 1 h, the chip was perfused at a low flow rate (*Q* = 0.5 μL min^−1^) with fresh culture media in the incubator until reaching confluence and prior to initiating the experiment.

*Microfluidics:* A microfluidic syringe pump (PHD 200, Harvard Apparatus, Cambridge, MA, USA) was used to perfuse the samples at physiological flow rates (*Q* = 0.5–1.0 μL min^−1^) and shear stress [8,14]. FITC-labeled latex tracer beads (Fluoresbrite^®^ YG Microspheres 0.75 μm, Polysciences, Inc.; 4 × 10^6^ beads mL^−1^) were used to experimentally measure the flow velocity profiles inside the channel. Three different fluids were used to characterize the hydrodynamic properties of the chip at room temperature, namely PBS, serum, and complete human blood. Collected blood was stabilized with 10% ethylenediaminetetraacetic acid (EDTA) (1 g EDTA in 10 mL ddH_2_O), immediately used after arrival, or maintained at 4 °C in the dark prior to use. Human blood samples were obtained from donors at the Blood and Tissue Bank (Barcelona, Spain), with written consent and in accordance with their Ethics Committee protocols. For microfluidic experiments, blood aliquots of 500 μL were allowed to equilibrate to room temperature before being injected into the microfluidic chip using 1 mL syringes. Special care was taken to prevent bubble formation (syringe and tubing) during blood injection by using the microfluidic pump.

*Staining:* A PBS 1× solution of Wheat Germ Agglutinin Alexa (WGA) Fluor^®^ 488 Conjugate (1 μg mL^−1^; ThermoFisher) was used to label the membrane of live endothelial cells) as recommended by the manufacturer. Briefly, a stock solution of 1 mg mL^−1^ WGA was prepared in PBS 1× and stored at −20 °C prior to use. Next, the stock solution was diluted into Hank’s balanced salt solution (HBSS) to obtain a working concentration of 1 μg mL^−1^. Cells were completely covered with the labeling solution and incubated for 10 min at 37 °C. Finally, the cells were washed with PBS and media.

*Data analysis:* The tracer FITC-labeled latex beads were tracked to measure the velocity of the fluid inside the microchannel and to compare the values with fluid dynamics simulations. The average velocity was measured as follows:

(1) $$\langle v\rangle =\frac{\sum_{i=1}^{n}v_{i\left(n-1\right)}}{n-1}$$

where *v_i_* is the instantaneous velocity between two consecutive images. The acquisition rate was set at 1 image/2 ms. The distance traveled by the tracer beads is *δ* = (Δx^2^ + Δy^2^)^1/2^. The angular displacement *λ* was calculated as follows:

(2) $$\varphi_{i}=asin\left(\frac{\Delta x_{i}}{\delta }\right)$$

where Δ*x_i_* is the lateral displacement of a tracer bead for each time point.

*Computational modelling and analysis*: COMSOL Multiphysics software (v5.0, Burlington, MA, USA) was used to numerically simulate and characterize blood flow and shear stress distribution within the microchannel. The simulations were done using human whole blood, serum, and water with similar properties as PBS. For blood, a Carreau-Yasuda model was used to mimic its non-Newtonian properties. We used a stationary physics study at *Q* = 0.5–1.0 μL min^−1^. The following parameters were used: ρ_H20_ = 1000 kg m^−3^, η_H2O_ = 1 cP or 0.000653 Pa s, ρ_serum_ = 906 kg m^−3^, η_serum_ = 1.1 cP, ρ_blood_ = 1060 kg m^−3^, η_blood_ = η_∞_ + (η_0_ − η_∞_) [1 + (λφ^2^] ^(*n*−1)/2^ (where η^∞^ = 0.0035 Pa s, η_0_ = 0.056 Pa s, λ = 3.313, and *n* = 0.3568). No-slip condition was set on all the microchannel boundaries, except for the outlets, where null pressure was imposed, and for the inlet, where the flow rate was fixed. Stationary simulation condition was set for the simulation.

Micro-particle image velocimetry (micro-PIV) analysis was performed as described in [15] using the PIV plug-in (implemented in ImageJ). Briefly, the displacement of red blood cells (RBCs) (vector and magnitude) was calculated by the normalized cross-correlation coefficient algorithm (template matching), so that an individual interrogation window was compared with a larger searching. This method compares the positions of the tracer particles within an individual interrogation window in two consecutive images to determine the average displacement. Finally, cell alignment analysis was performed using Tissue Analyzer [16]. Briefly, the raw phase-contrast image of the endothelialized channel was segmented and the resulting image used to measure flow-induced cell polarity/alignment. Finally, shear stress τwas estimated as follows: τ ≈ η (δv/δy), where η is the viscosity, and δv/δy is the velocity gradient.

## 3. Results

### 3.1. Hydrodynamic Flow Properties

**Figure 2:**
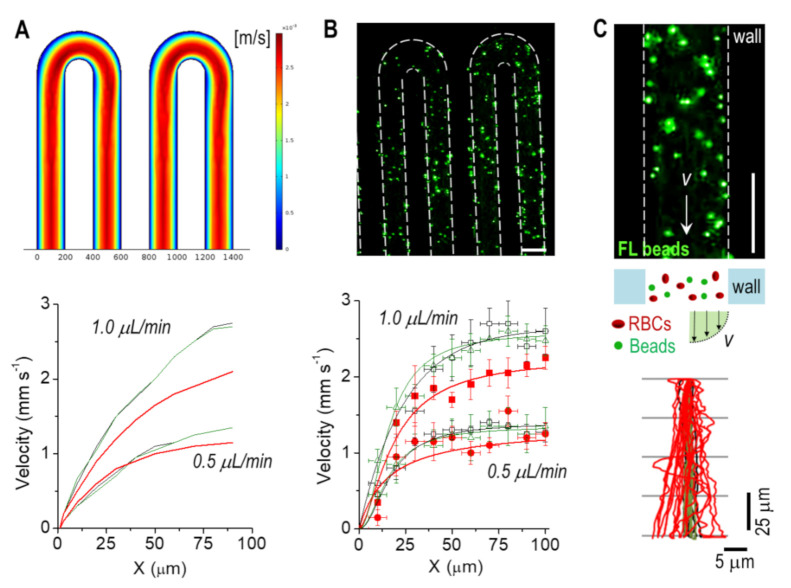
Hydrodynamics characterization of the vessel-on-a-chip. (**A**) Fluid dynamics simulation showing the velocity magnitude (Q = 1.0 μL min^−1^) and velocity profiles for PBS (in black), serum (in green), and blood (in red) at Q = 0.5 and 1.0 μL min^−1^. (**B**) (Upper) Fluorescence microscopy image of the vessel-on-a-chip. (Lower) Measured velocity profiles for all fluids and flow rates. (**C**) (Upper) Magnified image of the channel with tracer fluorescent beads. (Lower) Experimental measurement of flow trajectories used. Scale bar: 200 μm.

We developed a simple microfluidic device to study in vitro and in silico the hydrodynamics of blood flow at physiological flow rates (*Q* = 0.5 and 1.0 μL min^−1^). The microchannel had a serpentine morphology to facilitate the observation of blood flow and distinctive traits, and to study the hemodynamics of blood in a native-like (tortuous) environment (Figure 1). We first analyzed in silico the flow of blood, which showed a characteristic parabolic velocity profile (Figure 2A). The dynamic viscosity also showed a similar profile at the linear channel regions (Figure S2). At the bends of the serpentine though, a larger viscosity was obtained in the inner side of the channel. Experimentally, we measured blood hydrodynamics by adding fluorescent beads. This allowed us to accurately track the red blood cells (RBCs), which was particularly helpful at high hematocrit (Hct) %. The maximum velocity values (i.e., at the center of the channel) matched those from the simulation remarkably well (using the same conditions; Table S1) (Figure 2B). The high Hct % also suggested a large scattering of RBCs. To confirm this hypothesis, we next measured the interaction of beads with RBCs and, as control, in PBS and serum (FBS). For the latter, the beads followed a quite straight trajectory as expected (only small fluctuations were observed), with a higher velocity profile (at the center of the channel) compared to blood; simulation results also confirmed this observation (Figure 2A). In contrast, the interaction of the beads with RBCs caused a significant scattering of their trajectories (Figure 2C). We quantified the degree of this scattering by measuring the average scattering angle φ finding a similar value for the studied flow rates (φ_Q = 0.5_ = 1.2 ± 0.7° and φ_Q = 1_ = 1.3 ± 0.9°). Next, we used these values to estimate the maximum length *L* for a bead located in the middle of the channel to interact with the vessel wall; again, similar values were obtained (*L*_Q = 0.5_ = 4.8 ± 2.8 mm and *L*_Q = 1_ = 4.4 ± 3.0 mm). These lengths were much smaller than the total length of our channel (~ 200 mm), indicating that the interaction of the beads—RBCs/RBCs—had a significant impact on their dynamics (e.g., friction). This effect may be exploited by drug-loaded nanocarriers for their successful accumulation into the targeted region. Specifically, the ‘margination’ of carriers towards the vessel walls may facilitate their extravasation across the fenestrated endothelium described in tumoral endothelium, as is widely reported [17,18].

### 3.2. Vessel-On-A-Chip Platform: Reproducing Distinctive Traits of Vascular Tumors

#### 3.2.1. Biophysical Characterization

**Figure 3:**
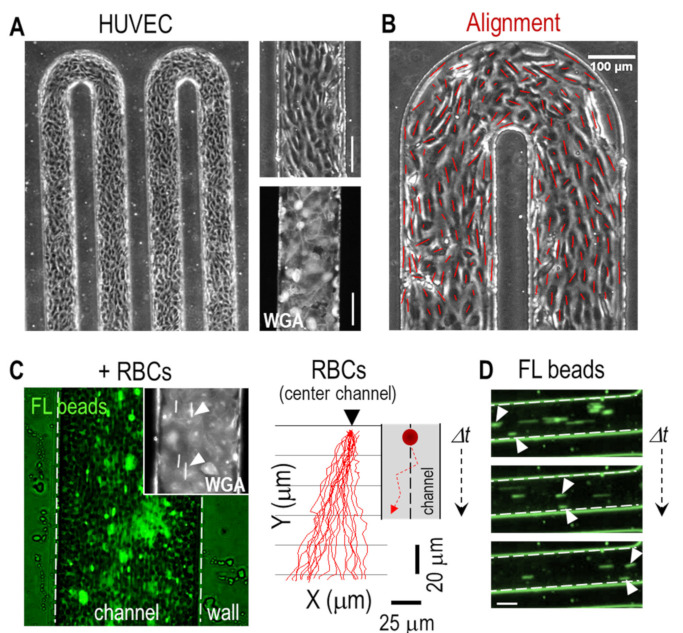
Vessel-on-a-chip with endothelial cells and red blood cells (RBCs). (**A**) (Left) Phase contrast microscopy image showing the serpentine microchannel coated with a confluent layer of HUVEC after 3–4 days in culture; (Right) detailed images (phase contrast and Wheat Germ Agglutinin Alexa (WGA) staining) showing the uniformity of the cell layer). (**B**) Flow-induced cell alignment (cell orientation is indicated in dark red). (**C**) (Left) Overlay of fluorescent and brightfield microscopy images showing the flow of RBCs and fluorescent beads along the channel (Movie S1). Inset, WGA-stained HUVEC cells with fluorescent beads (white arrowheads) flowing along the channel. (Right) Trajectory of fluorescent beads along the endothelium-like microchannel showing a significant degree of scattering due to cellular collisions (0.5 μL min^−1^). For the analysis, we selected the beads flowing in the center of the channel. (**D**) Time sequence of flowing fluorescent beads. The bead next to the channel wall displays a lower velocity compared to the one in the center (white arrowheads). Scale bars: 100 μm.

We next coated the walls of the microchannel with endothelial cells to reproduce the cellular content of a native human blood vessel (see Methods). Figure 3A shows that HUVEC formed a complete endothelium along the channel walls as demonstrated by both the phase-contrast image and by the staining of the endothelial cells with WGA. The engineered endothelium was continuously perfused with media (*Q* = 0.5 μL min^−1^), inducing a shear stress that polarized and aligned the cells in the direction of the flow compared to static condition (Figure 3B and Figure S4); larger flow rates (Q = 1 μL min^−1^) did not significantly alter the degree of alignment. We estimated a wall shear stress τ value ≈ 0.1 Pa; considering the size of the microchannel and flow rates, it was representative of a physiological condition for venules/veins vasculature [8,19]. Similarly, a shear stress of τ ≈ 0.2 Pa was obtained for *Q* = 1 μL min^−1^, also representative of the same type of vasculature. We next injected whole blood spiked with fluorescent beads into the vessel-on-a-chip (Figure 3C and Movie S1). Again, we observed a large scattering of the beads due to the high Hct and to the synergistic interaction of the beads with the RBCs/endothelial cells. Similar to the non-coated condition, we observed that both the RBCs and the beads located next to the vessel wall, circulated at lower velocities (Figure 3D*).* We also investigated the trajectories of RBCs at the bends of the channel and found larger speed values compared to the linear regions. Additionally, RBCs traveled at a significantly larger speed at the inner side compared to the outer side of the bend (Figure S3 and Movie S2); this effect was also observed in the simulation (Figure 2A).

#### 3.2.2. Thrombus and Hemorrhage Formation

Vascular tumors are characterized by a perturbed blood flow that increases the risk of developing coagulopathies, such as thrombosis (i.e., blood clot formation) or thrombocytopenia (i.e., serious bleeding due to leaky vessels) [20]. Therefore, the recapitulation of blood clot formation and hemorrhages within a vessel-on-a-chip may result in a useful tool to improve our knowledge about the hemodynamics of blood, as well as to investigate the etiology of the disease. In this regard, we next developed a reductionistic *thrombus* and *hemorrhage-on-a-chip* platform that reproduced these distinctive features.

**Figure 4:**
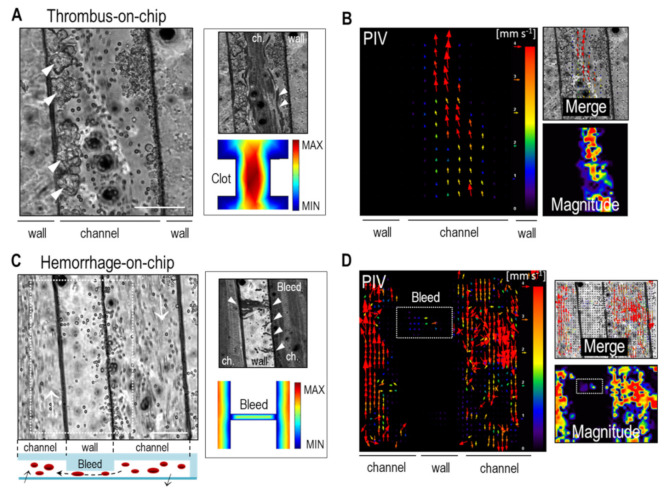
Thrombus and hemorrhage formation. (**A**) (Left) Thrombus-on-a-chip: formation of blood clots (white arrowheads) on the fibronectin-coated channel walls (Movie S3). (Right) Temporal projection and fluid flow simulation of RBCs circulation across a thrombi region. White arrowheads highlight the acceleration/deceleration of RBCs. (**B**) The generated constriction increased the flow velocity of RBCs as demonstrated by the micro-PIV analysis (vectorial image, merge, and magnitude measurement). (**C**) (Left) Hemorrhage-on-a-chip: formation of a leaky vessel mimicking vascular tumor-related bleeding from detached—void—PDMS wall regions and transit towards the adjacent channel. (Right) Temporal projection showing the bleeding regions and trajectories of the RBCs (white arrowheads) (Movie S4). The fluid flow simulation (modelling a bleed region) shows the transit of RBCs across adjacent channels. (**D**) The micro-PIV plots show the motion of RBCs inside and outside the channel. Scale bars: 100 μm.

In healthy normal vasculature, the endothelium has a non-adhesive and natural anti-thrombotic effect for the correct transport of RBCs. In contrast, during pathological conditions, the local expression and secretion of adhesive proteins by the endothelium promotes the formation of thrombi. To reproduce this condition, and taking into consideration that the blood contained the anticoagulant EDTA that avoided clot formation, for this experiment we replaced the endothelial layer by a clot-activating fibronectin coating (20 μg mL^−1^; see Methods) to promote the formation of clot-like RBCs aggregates. Future refinement to this work may include the integration of a complete endothelium and the use of non-treated whole blood. The resulting reductionistic vessel-on-a-chip was perfused with (diluted) human blood at a constant low flow rate (*Q* = 0.5 μL min^−1^) to favor adhesion of RBCs, and observed the rapid and massive formation of blood clot-like aggregates (Figure 4A and Movie S3). The number and size of the formed aggregates increased with time and ranged from few tenths of μm^2^ to larger blood clots that bridged the opposite walls of the channel. We observed that in a bulky thrombus, blood velocity increased dramatically. This increased velocity was also observed in the simulations and resulted from the non-Newtonian properties of blood, which suffers from shear thinning and reduced viscosity (Figure 4A). The perturbed flow also caused a stochastic rolling of RBCs, as described elsewhere [21]. We next investigated the hydrodynamics of RBCs in regions near thrombus formation by real-time optical microscopy and label-free micro-particle image velocimetry (micro-PIV). We observed from the temporal projection that, between the clots located next to the wall, RBCs became trapped with clear acceleration and deceleration regimes (Figure 4A). Next, micro-PIV analysis showed as expected a velocity field where RBCs increased their speed in the more constricted regions, also in agreement with the fluid flow simulations (Figure 4B—PIV and merge). From the displacement field, we also obtained the vector magnitude plot (normalized), which showed a higher velocity of RBCs in the thrombi regions. These values were in agreement (same order of magnitude) with the measured velocity profiles (Figure 2B—blood), but with an approximately twofold increase for the constricted—thrombi—regions of the channel. We found small fluctuations in the measured velocity profiles when flowing in a crowded environment, which depended on the analyzed region (presence and clot size) and time (local concentration of RBCs; equivalent to Hct %). Note that formally speaking, the formed thrombus cannot be denoted as ‘clots’ as the clotting cascade is negated by EDTA. The observed ‘clots’ may be therefore considered as multi-aggregates of RBCs and proteins/platelets. In any case, the generation of clot-like structures can also reproduce the thrombi formation of vascular tumors. Overall, despite this inaccuracy, our platform and data may be of great interest to better understand the biophysical behavior of blood in clot-containing microvessels, and to elucidate the blood transport mechanisms in physiological and pathological conditions, in particular, in vascular-associated diseases, such as thrombosis. Similarly, despite its simplicity, this thrombus-on-a-chip model may exhibit a vast array of applications to investigate the factors (e.g., fibrin, fibrinogen, platelets, and others) triggering the formation of blood clots as well as the effect of novel anti-coagulant or anti-platelet agents in a well-controlled manner.

We next investigated the hemodynamics of bleeding using the reductionistic hemorrhage-on-a-chip platform (Figure 4C). For this, we created non-adhesive regions along the channel walls (by fine tuning the adhesion of the elastomer with the glass) of around 20 μm in length to artificially increase the permeability of the channels (i.e., enhanced fenestrated channel), thus reproducing a defect (or injury) in the vessel without the need of coating the channels with endothelial cells. In this case though, we did not coat the channel with fibronectin to prevent RBCs aggregation. Upon perfusion (*Q* = 0.5 μL min^−1^), RBCs continued to flow mainly through the engineered vessels. However, small ´hemorrhages´ along the microchannel were observed (Figure 4C and Movie S4). The engineered small non-adhesive openings together with serpentine morphology induced a Venturi-powered and negative pressure-driven extravasation/suction of RBCs that reproduced in vitro a small hemorrhage. A small percentage of these cells (<5%) were captured in the void detached area, whereas the remaining RBCs transited across it to return back in the adjacent channel (Movie S4). Indeed, the time-sequence projection shows these hemorrhage regions and the transit of RBCs (Figure 4C, white arrowheads). The fluid flow simulation modeling the bleeding region also showed the slow transit of RBCs (Figure 4C). Finally, we used micro-PIV analysis to further describe the dynamics of the bleeding site. Again, the obtained results showed the intra- and extra-vasation of RBCs but at a reduced velocity compared to the main flow along the channel (Figure 4D). We experimentally measured the average speed of RBCs across the bleeding region finding a value of <v*_bleed_*> = 1.14 ± 1.20 mm s^−1^, in agreement with the PIV results. Finally, the experimental flow velocity patterns showed a complex and heterogeneous dynamic behavior compared to the simulated ones, highlighting the impact of bleeding in the flow dynamics of RBCs.

Interestingly, we also found the formation of small clots next to the engineered bleeding injury (Figure S5 and Movie S5). However, due to the absence of an actual endothelium, we can only speculate that this is due to a direct platelet activation and fibrin accumulation reproducing physiological coagulation. To confirm this, future refinement would be needed to assess the actual mechanism and molecular characterization of clot formation by the addition of endothelial cells (and use of non-treated blood). Indeed, some works using microfluidics have shown the pro-coagulant function of the endothelium that is required for hemostasis [22].

Taken together, these results show the applicability of our vessel-on-a-chip system to model thrombi formation and hemorrhage in a reductionistic and controllable manner. Despite its simplicity, this is one of the few models of hemorrhage on-chip, paving the way towards its future applicability in pre-clinical modeling and drug discovery/screening.

## 4. Discussion

During tumor development, the (micro-) vascular system suffers from severe morphological changes, which perturbs the normal flow of blood causing a life-threatening condition. Besides cancer, vessel anomalies have been described in a myriad of diseases. Indeed, around 25% of all deaths worldwide are related to acute problems in blood flow, such as thrombosis, embolism, or bleeding. Investigating the behavior of blood in confined environments may help to assess the mechanistic determinants of vascular-related diseases, to design new biomedical devices, to discover novel nanomedicines, or to develop more effective diagnostic tools [23].

Blood flow has been largely studied, in particular in large arteries [24,25,26,27]. However, less is known about its behavior in smaller vessels despite the number of investigations describing the use of microfluidics with blood. In this work, we showed how microfluidics technology, biophysical analysis, and computational simulations can be employed to reproduce distinctive traits of (micro-) vascular-related diseases in well-controlled dynamic conditions. In particular, we developed a reductionistic vessel-on-a-chip model to study key characteristic features of infantile vascular tumors, a rare disease with limited knowledge and treatment. With this aim, we used a diverse set of biophysical tools to analyze the behavior of blood within a biomimetic tortuous microfluidic channel. Using fluorescent beads as tracers, we found a large scattering that forced the beads to interact with the vessel wall, an effect that can be exploited to improve the design of carriers for the local delivery of therapeutics. We also found that RBCs accumulated in the central streamline of the channel producing a cell-free marginal layer next to the channel wall [28]. This other effect has been already described for RBCs flowing in microvessels smaller than 300 μm in width [23].

Current thrombus and bleeding studies are based on complex assays, in particular, in vivo animal injury models. These models have provided valuable insights about the mechanism of hemorrhage and clotting, but the obtained results may not be directly translated to humans, besides being ethically controversial and very expensive [29]. The inherent advantages of microfluidics and organ-on-a-chip technology allow the investigation of these vascular injury events in a well-controlled dynamic environment to assess the biological, biochemical, and biophysical components of the native scenario [22,30,31]. To that end, we developed reductionist in vitro models of thrombus- and hemorrhage-on-a-chip, which were capable of promoting the massive formation of (human) blood clots or bleeding. These characteristic features can be additionally exploited to assess additional thrombotic events in other life-threatening diseases, such as in COVID−19 [32], or to assess the dynamics of blood-related pathologies, such as anemia [33]. Despite the simplicity of the developed platform, it displays several advantages compared to other assays, including a high flexibility, easy manipulation, low cost, and real-time imaging of the pathological processes. Additionally, the model can be easily adapted in terms of cell content (endothelial cells—blood and lymphatic), blood subtypes (from healthy individuals to patients with vascular pathologies), biophysical parameters (e.g., physiological and pathological flow rates—shear stress), molecular content (e.g., addition of drugs and pharmacological compounds), Hct %, which is lower in infants (and therefore, it must be carefully adjusted if modelling infantile vascular tumors), or *Q*, which increases with increasing age [34].

In this work, we have established the technological basis of a microfluidic platform compatible with massive thrombi formation and bleeding in an extremely simple and rapid manner, enabling its use by non-experienced users that aim to investigate this type of pathophysiological events. Finally, even though most of the observed events have already been reported, this work showcases the versatility of our platform and its applicability to reproduce different dynamic features of vascular-related diseases in a simple and reductionistic manner. Future refinement to our work though may include increasing the physiological relevance of this platform by, e.g., (I) the integration of both thrombus- and hemorrhage-on-a-chip models lined with endothelial cells to permit the measurement of key clinical parameters, such as bleeding time, the assessment of (anti-) coagulant formulations, or the assessment of its health and coverage in pathological conditions; (II) update the geometry of the microchannels to better mimic the native vessel morphology; (III) use of hydrogel instead of solid PDMS to better mimic the native microenvironment [35]; and (IV) use of fresh blood without anti-coagulants. This will result in a better understanding of the blood flow phenomena for an improved prevention, diagnosis, and treatment of vascular diseases, and in particular, for infantile vascular tumors.

## 5. Conclusions

In this work, we showed how microfluidics and biophysical analysis can be employed to reproduce and investigate distinctive features of vascular-related diseases, and in particular, vascular tumors. Despite being rare in childhood, it is of upmost importance to develop new and simple tools to study dynamic events characteristic of this disease. In this regard, vessel-on-a-chip systems can provide valuable insights about blood hemodynamics, and in the future, may contribute to elucidate how abnormal flow influences the onset of the pathology, and vice versa, how the growth of the tumor perturbs blood hemodynamics. Indeed, we analyzed the dynamics of human blood in different situations characteristic of rare vascular tumors. Of particular relevance are the works related to thrombus- and hemorrhage-on-a-chip, which may be useful for studying the formation of blood clots and bleeding in a simple and rapid manner. Overall, this work establishes the technological basis of a versatile platform capable of reproducing the distinctive traits occurring in (infantile) vascular tumors. This system may be utilized to investigate the dynamic events characteristic of vascular-related diseases or for the screening of novel pharmacological formulations, among others.

## Authors Contributions

Conceptualization: D.C., L.A., J.S.; investigation: M.L., R.P., and D.C.; visualization: M.L., N.F.-G. and D.C.; data analysis: M.L., R.P. and D.C.; theoretical description: M.L.; writing and editing: M.L., R.P., N.F.-G., L.A., J.S. and D.C.; overall supervision and funding acquisition: L.A., J.S. and D.C. All authors have read and agreed to the published version of the manuscript.

## Data Availability

The data presented in this study are available on reasonable request from the corresponding author.

## Supplementary Materials

The following data are available online: https://www.mdpi.com/article/10.3390/bioengineering8060081/s1. Table S1—Velocity values (simulated vs. experimental). Figure S1—The human vasculature in healthy and tumor conditions. Figure S2—Dynamic viscosity simulation. Figure S3—Characterization of the hydrodynamics at the serpentine bend. Figure S4—Cell alignment in static and flow conditions. Figure S5—Formation of coagulation in a hemorrhage-on-a-chip model. Movie S1—Hemodynamics of fluorescent-labeled tracer beads and RBCs in the vessel-like channel. Movie S2—Hemodynamics in curved channel. Movie S3—Thrombus-on-a-chip model. Movie S4—Hemorrhage-on-a-chip model. Movie S5—Combined thrombus and hemorrhage-on-a-chip model.
