# Supplementary material for: Versatile Vessel-on-a-Chip Platform for Studying Key Features of Blood Vascular Tumors
Source: Bioengineering (Basel). 2021 Jun 9;8(6):81. doi: 10.3390/bioengineering8060081 (PMC8226980; doi:10.3390/bioengineering8060081)
Supplement: Supplementary file 1 [file bioengineering-08-00081-s001.zip › Caballero_et_al_Supporting_Information2.pdf]

SUPPORTING INFORMATION

Versatile Vessel-On-A-Chip Platform for Studying Key Features of Blood Vascular Tumors

Marina Llenas [1,2,†], Roberto Paoli [1,2,3], Natalia Feiner-Gracia [1], Lorenzo Albertazzi [1,4,*], Josep Samitier [1,2,3,*] and David Caballero [1,2,3,*,‡]

1 Institute for Bioengineering of Catalonia (IBEC), The Barcelona Institute of Science and Technology (BIST), Baldiri Reixac 15–21, 08028 Barcelona, Spain; mllenas@icmab.es (M.L.); rpaoli@ibecbarcelona.eu (R.P.); nfeinerg@gmail.com (N.F.-G.)

2 Department of Electronics and Biomedical Engineering, University of Barcelona, C. Martí i Franqués 1, 08028 Barcelona, Spain

3 Centro de Investigación Biomédica en Red en Bioingeniería, Biomateriales y Nanomedicina (CIBER-BBN), 28029 Madrid, Spain

4 Department of Biomedical Engineering and Institute of Complex Molecular Systems (ICMS), Eindhoven University of Technology, 5612AZ Eindhoven, The Netherlands

* Correspondence: l.albertazzi@tue.nl (L.A.); jsamitier@ibecbarcelona.eu (J.S.); dcaballero@i3bs.uminho.pt (D.C.)

† Current address: Institut de Ciència de Materials de Barcelona (ICMAB-CSIC), Campus de la UAB, 08193 Bellaterra, Barcelona, Spain

‡ Current address: 3B's Research Group, I3B's—Research Institute on Biomaterials, Biodegradables and Biomimetics; and ICVS/3B's—PT Government Associate Laboratory, University of Minho, 4805-017 Barco, Guimarães, Portugal

Supplementary Tables

Table S1. *Velocity values (simulated vs. experimental).*

Q (µL min^{-1})	**PBS / Serum** *simulated* (mm s^{-1})	**PBS** *experimental* (mm s^{-1})	**Serum** *experimental* (mm s^{-1})	**Blood** *simulated* (mm s^{-1})	**Blood** *experimental* (mm s^{-1})
0.5	1.39	1.38 ± 0.07	1.34 ± 0.25	1.21	1.27 ± 0.17
1.0	2.76	2.71 ± 0.27	2.64 ± 0.12	2.10	2.27 ± 0.12

Supplementary Figures

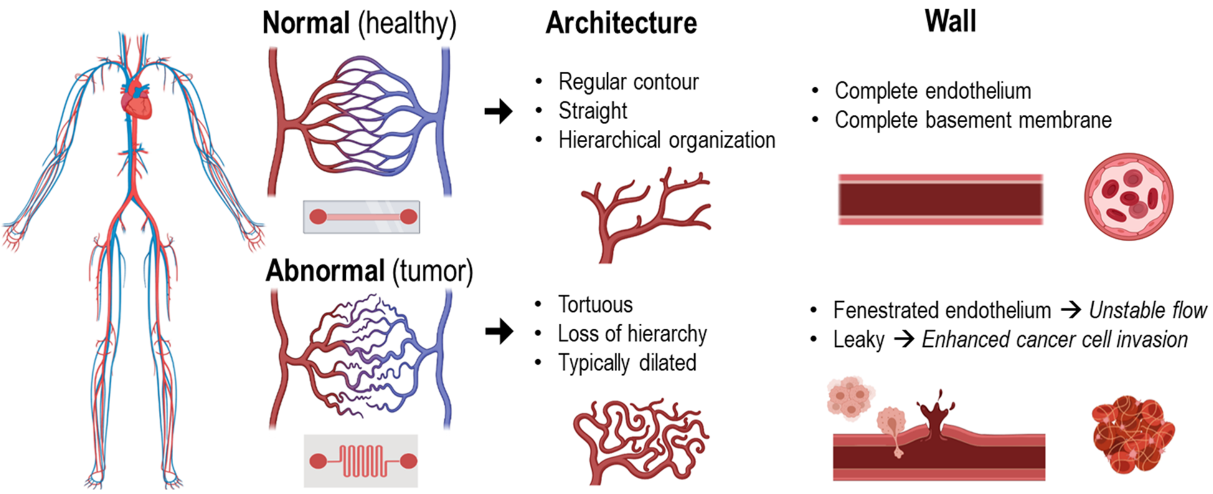

Figure S1. The human vasculature in healthy and tumor conditions. *(Left)* Illustration of the human body vasculature (arteries and veins), and detailed scheme of normal (healthy) and abnormal (tumor) capillary bed and associated microfluidic chip models. *(Right)* Summary of the main characteristics (vessel architecture and wall) of healthy vs. tumor vasculature. Created with Biorender.com.

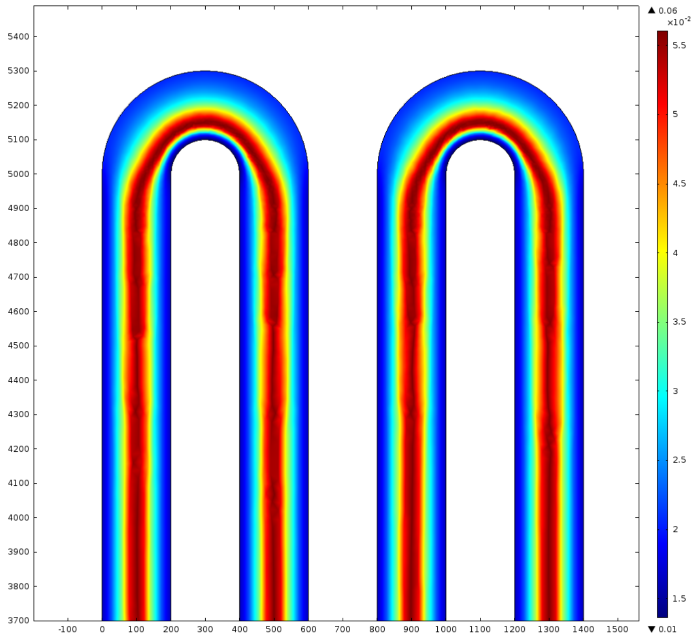

Figure S2. Dynamic viscosity simulation (in [Pa s]).

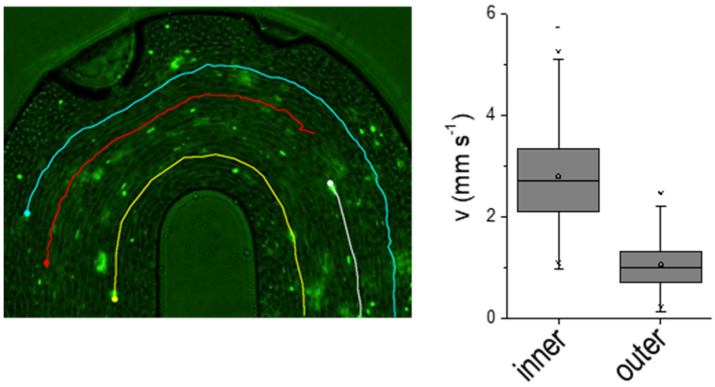

Figure S3. Characterization of the hydrodynamics at the serpentine bend. *(Left)* Tracking of tracer beads within the microchannel bend containing RBCs. *(Right)* Average velocity at the inner and outer side of the channel bend.

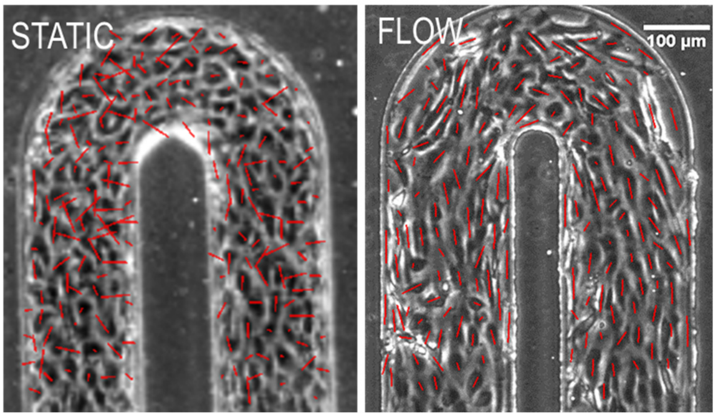

Figure S4. Cell alignment. *(Left)* static; *(Right)* flow conditions.

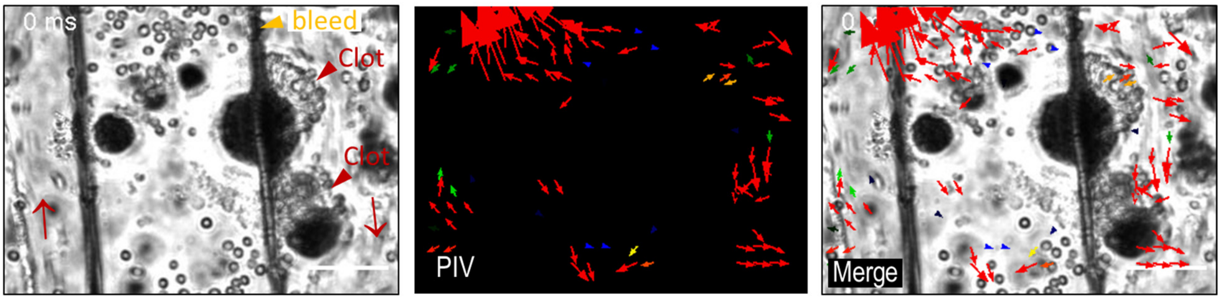

Figure S5. Formation of coagulation in a hemorrhage-on-a-chip model. *(Left)* Microfluidic chip highlighting the bleeding (in yellow) and coagulation (clot; in red) regions. *(Mid and Right)* PIV analysis (see also Movie S4). Scale bars: 100 µm.

Supplementary Movie Legends

Movie S1 – Hemodynamics of fluorescent-labelled tracer beads and RBCs in the vessel-like channel. Acquisition rate: 1 image/2 ms. Scale bar: 100 µm.

Movie S2 – Hemodynamics in curved channel. Acquisition rate: 1 image/2 ms. Scale bar: 100 µm.

Movie S3 – Thrombus-on-a-chip model. Acquisition rate: 1 image/2 ms. Scale bar: 100 µm.

Movie S4 – Hemorrhage-on-a-chip model. Acquisition rate: 1 image/2 ms. Scale bar: 100 µm.

Movie S5 – Combined thrombus and hemorrhage-on-a-chip model. Acquisition rate: 1 image/2 ms. Scale bar: 100 µm.
